# ALZpath pTau217: Multi‐cohort performance, cross‐assay comparison, and clinical launch for the identification of Alzheimer’s Disease pathology

**DOI:** 10.1002/alz.094376

**Published:** 2025-01-09

**Authors:** Lauren E Chaby, Jacob Borello, Timothy Vaughan, Stuart D Portbury, Hans Frykman, Anna Mammel, Mary Encarnacion, Geidy E Serrano, Rachael E Wilson, Lianlian Du, Erin M. Jonaitis, Sterling C. Johnson, Andreas Jeromin

**Affiliations:** ^1^ ALZpath, Inc., Carlsbad, CA USA; ^2^ ALZpath, Carlsbad, CA USA; ^3^ BC Neuroimmunology lab, Vancouver, BC Canada; ^4^ Neurocode USA Inc., Bellingham, WA USA; ^5^ Banner Sun Health Research Institute, Sun City, AZ USA; ^6^ Wisconsin Alzheimer’s Institute, University of Wisconsin School of Medicine and Public Health, Madison, WI USA; ^7^ University of Wisconsin, Madison, WI USA; ^8^ University of Wisconsin School of Medicine and Public Health, Madison, WI USA; ^9^ School of Medicine and Public Health, University of Wisconsin‐Madison, Madison, WI USA

## Abstract

**Background:**

Blood‐based AD biomarker tests will be essential clinical tools to provide accessible and affordable screening and monitoring for AD disease‐modifying therapeutics (DMT) as well as advancing overall clinical care. Tau phosphorylated at position 217 (pTau217) is considered to have the highest accuracy in identifying Alzheimer’s disease (AD) pathology using blood. We describe a multi‐cohort evaluation of the Simoa ALZpath pTau217 assay in plasma, including memory clinic patients, as well as performance in the context of other commercially available pTau217 assays. Finally, we discuss the clinical launch of ALZpath pTau217 including pTau217 reference intervals and clinical cutoffs in the context of other pTau217 assays.

**Method:**

The ALZpath pTau217 assay is an ultra‐sensitive blood‐based test developed on the semi‐automated single‐molecule array Simoa platform; it is the first commercially available pTau217 test and has been used in over 40 cohort studies with over 50,000 sample tests. To enable clinicians to leverage ALZpath pTau217 to inform care decisions, ALZpath Dx has been validated in partnership with Neurocode CLIA‐certified laboratory.

**Result:**

We discuss multi‐cohort ALZpath pTau217 findings for the prediction of amyloid and tau burden, measured using PET imaging and post‐mortem histology. In a pTau217 cross‐assay evaluation, ALZpath pTau217 had the strongest relationship with amyloid load as measured by (PiB) PET in a population of healthy and MCI individuals in comparison with other blood‐based AD biomarker assessments including the integration of multiple biomarkers. We discuss pTau217 reference intervals derived from multiple normative populations and compare pTau217 levels detected across multiple pTau217 assays, demonstrating that reference intervals are currently highly assay specific, highlighting the importance of efforts underway to develop a pTau217 reference standard. Finally, we report the clinical launch of ALZpath Dx, including pTau217 clinical cutoffs derived from cross‐cohort analyses, and the implications for the dementia clinical care system.

**Conclusion:**

Multi‐cohort findings support ALZpath pTau217 as a high performing diagnostic biomarker for Alzheimer’s disease with high accuracy in determining amyloid burden across all stages of AD continuum. ALZpath pTau217 performance capabilities can support timely AD identification and intervention and facilitate scalable DMT implementation.

